# Placental malperfusion in response to intrauterine inflammation and its connection to fetal sequelae

**DOI:** 10.1371/journal.pone.0214951

**Published:** 2019-04-03

**Authors:** Solange N. Eloundou, JiYeon Lee, Dan Wu, Jun Lei, Mia C. Feller, Maide Ozen, Yan Zhu, Misun Hwang, Bei Jia, Han Xie, Julia L. Clemens, Michael W. McLane, Samar AlSaggaf, Nita Nair, Marsha Wills-Karp, Xiaobin Wang, Ernest M. Graham, Ahmet Baschat, Irina Burd

**Affiliations:** 1 Integrated Research Center for Fetal Medicine, Division of Maternal Fetal Medicine, Department of Gynecology and Obstetrics, Johns Hopkins University, School of Medicine, Baltimore, MD, United States of America; 2 The Russell H. Morgan Department of Radiology and Radiological Science, Johns Hopkins University, School of Medicine, Baltimore, MD, United States of America; 3 Division of Neonatology, Department of Pediatrics, Johns Hopkins University, School of Medicine, Baltimore, MD, United States of America; 4 Department of Pathology, King Abdulaziz University, Jeddah, Kingdom of Saudi Arabia; 5 Department of Environmental Health and Engineering, Johns Hopkins University, School of Public Health, Baltimore, MD, United States of America; 6 Department of Population, Family and Reproductive Health, Center on Early Life Origins of Disease, Johns Hopkins University, School of Public Health, Baltimore, MD, United States of America; 7 Fetal Therapy, Department of Gynecology and Obstetrics, Johns Hopkins University, School of Medicine, Baltimore, MD, United States of America; University of Wisconsin - Madison, School of Veterinary Medicine, UNITED STATES

## Abstract

Exposure to intrauterine inflammation (IUI) is associated with short- and long-term adverse perinatal outcomes. However, little data exist on utilizing placenta to prognosticate fetal injury in this scenario. Our study aimed to utilize imaging modalities to evaluate mechanisms contributing to placental injury following IUI exposure and correlated it to concomitant fetal brain injury. CD1 pregnant dams underwent laparotomies and received intrauterine injections of either lipopolysaccharide (LPS; a model of IUI) or phosphate-buffered saline (PBS). *In utero* ultrasound Doppler velocimetry of uterine and umbilical arteries and magnetic resonance imaging (MRI) of placental volumes with confirmatory immunohistochemical (vimentin) and histochemistry (fibrin) analyses were performed. ELISA for thrombosis markers, fibrinogen and fibrin was performed to analyze thrombi in placenta. Fetal brain immunohistochemistry was performed to detect microglial activation (ionized calcium-binding adaptor molecule 1, Iba1). On ultrasound, LPS group demonstrated elevated resistance indices, pulsatility indices and a greater occurrence of absent end-diastolic flow in the umbilical and uterine arteries. In the fetus, there was an increased cardiac Tei indices in the LPS group. MRI revealed decreased volume of placenta in the LPS group associated with placental thinning and placental endothelial damage on immunohistochemistry. Decreased fibrinogen content and more thrombi staining in placenta exposed to maternal LPS indicated the hypercoagulability. Furthermore, the expression of Iba1was significantly associated with placental thickness (r = -0.7890, Pearson correlation coefficient). Our data indicate that IUI can trigger events leading to maternal placental malperfusion and fetal vessel resistance, as well as predispose the developing fetus to cardiac dysfunction and brain damage. Furthermore, our data suggest that prenatal ultrasound can be a real-time clinical tool for assessing fetal risk for adverse neurologic outcomes following the potential IUI exposure.

## Introduction

Intrauterine inflammation (IUI) during pregnancy is associated with numerous adverse perinatal outcomes initiated as part of the fetal inflammatory response syndrome (FIRS) [1,2,3]. FIRS contributes to fetal mortality, as well as a spectrum of short-term and long-term morbidities, including bronchopulmonary dysplasia and neurologic injury[3]. Of the approximately 6 million perinatal deaths each year, preterm birth associated with IUI may contribute to up to 27% of the mortality[4,5,6,7,8]. Timely diagnosis and prognostication of fetal injury may allow early intervention to prevent further sequelae of IUI exposure.

A major limitation of human studies is that placenta is obtained after delivery, thus there is a reduced ability to study placenta during pregnancy. Animal models overcome this limitation. Recently, researchers have demonstrated that ultrasound (US), with and without concomitant use of magnetic resonance imaging (MRI), may serve as a useful diagnostic tool for the detection of fetal neurological and placental complications during pregnancy [9,10]. However, placental imaging changes following IUI are not well-characterized, and whether these changes are correlated with fetal brain injury is not known.

In this study, we hypothesized that IUI results in hemodynamic and structural changes in placenta, which in turn lead to fetal sequelae. Specifically, we sought to determine whether placental changes occur as a result of thrombus formation and whether these changes are correlated with fetal microglial activation.

## Materials and methods

### Animal preparation

All care and treatment procedures were approved by the Animal Care and Use Committee of Johns Hopkins University (Hopkins-IACUC Protocol No. MO14M326) and were carried out in accordance with institutional standards. Pregnant CD-1 mice (Charles River Laboratories, Wilmington, MA, USA) were used for the study. On embryonic day 17 (E17), pregnant dams were subjected to a well-established model of IUI as per a previously described protocol[11,12,13,14]. Briefly, mice were placed under isoflurane anesthesia (Baxter # NDC 10019-360-60, Deerfield, IL, USA) before undergoing a mini-laparotomy; 25 μg of lipopolysaccharide (LPS, Sigma Aldrich, St Louis, MO, USA from E. coli O55:B5, n = 56) in 100 μl phosphate-buffered saline (PBS) or vehicle (PBS, n = 43) were injected between the first and second embryos of the lower right uterine horn. Routine laparotomy closure was performed, and the dams recovered.

### Doppler examination

Doppler examination was performed for the uterine artery (UtA), placental and fetal sides of the umbilical artery (UA), and fetal Myocardial Performance Index (MPI/Tei) six hours after LPS-exposure in the IUI group and PBS-exposure in the control group. Briefly, general anesthesia was induced by inhalation of 5% isoflurane and oxygen at 3 L/minute and maintained with 1.5% isoflurane and oxygen at 2 L/minute. Doppler waveforms of the UtA were obtained at the level where the main UtA branches from the internal iliac artery. The systolic/diastolic (S/D) ratio, resistance index (RI), pulsatility index (PI) and the presence of early diastolic notch were evaluated in UtA. Doppler waveforms of the placental and fetal sides were obtained at the placental cord insertion sites and fetal abdominal cord insertion sites, respectively. RI, PI and the presence of absent end-diastolic flow (AEDF) were evaluated in the UA. S/D ratio was calculated as (peak systolic velocity/end-diastolic velocity). RI was calculated as [(peak systolic velocity–end-diastolic velocity)/peak systolic velocity]. PI was calculated as [(peak systolic velocity–end-diastolic velocity)/mean velocity]. To measure the Tei index, Tissue Doppler Imaging (TDI) of the mitral annulus was obtained from the apical four-chamber view. The Tei index was calculated as [(isovolumic contraction time + isovolumic relaxation time)/ejection time]. Doppler waveforms of umbilical arteries and Tei index was obtained from both sides of the uterus (1 to 2 fetuses on the right, 1 to 2 fetuses on the left). Doppler waveforms of uterine arteries were measured on the right and left side. The right and left Doppler waveforms were not different. All Doppler waveforms were taken at an angle as close as possible to 0°. Vevo 770 (VisualSonics, Toronto, Ontario, Canada) was utilized for the Doppler evaluation.

### MRI imaging

*In utero* MRI was performed six hours after LPS exposure. Pregnant dams were anesthetized with 1% isoflurane, along with air and oxygen, at a 3:1 ratio via a vaporizer. A horizontal 11.7T MRI scanner (Bruker Biospin, Billerica, MA, USA), with a 72-mm diameter quadrature transmitter coil and an eight-channel phased array rat body coil, was utilized. The multislice T2-weighted images were acquired with a Rapid Acquisition with Relaxation Enhancement (RARE) sequence using the following imaging parameters: field-of-view (FOV) = 40 x 32 mm, encoding matrix = 320 x 256 giving to an in-plane resolution of 0.125 x 0.125 mm, 0.5 mm slice thickness and 60 slices, echo time (TE)/repetition time (TR) = 24/5000, eight echo trains, four signal averages, and scan time of 11 minutes. The placental volumes were obtained by manual delineation of the placenta based on the T2-weighted images.

### Histochemistry and immunohistochemistry (IHC)

Six hours after injection, isolated whole fetal brains and placentas from another set of mice with IUI–not those imaged–were fixed overnight at 4°C in 4% paraformaldehyde (PFA) (Affymetrix Inc., Cleveland, OH, USA). Tissue was then immersed in 30% sucrose (Sigma-Aldrich, St. Louis, MO, USA). Using Leica CM1950 cryostat (Leica Biosystems Inc., Buffalo Grove, IL, USA), the specimens were cut at 20 μm thickness and mounted on positively charged slides (Fischer Scientific). Fetal brains were cut coronally, and placentas were cut transversally. Two fetuses from the lower right horn and two fetuses from lower the left horn were sampled per each dam. Since the animals were treated in pregnancy, each dam represented an *n* of 1. The measurement of the placental thickness (total as well as maternal and fetal components) was done across the central cut surface of the placenta.

Routine hematoxylin and eosin (H&E) staining was performed on placentas to evaluate the morphological changes. For IHC, slides were incubated in PBS solution containing 0.05% Triton X-100 (Sigma-Aldrich) and 5% normal goat serum (Invitrogen, Carlsbad, CA, USA) for 30 minutes. Placental tissues were incubated with endothelial markers, rabbit anti-vimentin antibody (1:200, Abcam, Cambridge, MA, USA) and rat anti-CD31 (1:200, Abcam). Ionized calcium-binding adaptor molecule 1 (Iba1) is a marker of fetal brain injury [15]. Fetal brains were incubated with Iba1 antibody (1:400, Wako Chemicals, Richmond, VA, USA) [16]. To specify Iba1 staining, Ig G negative control was used in place of a primary rabbit antibody. Donkey anti-rabbit Alexa Fluor 568 and donkey anti-rat Alexa Fluor 488 fluorescent secondary antibodies were utilized (1:500, Life Technologies, Grand Island, NY, USA) along with DAPI counter-staining. All images analyzed were obtained from Zeiss AxionPlan 2 Microscope System (Jena, Germany). The images for placental thickness measurements were taken under 5x magnification. IHC images of vimentin, CD31 and Iba1 quantification were obtained under 20x magnification. Five area of fields were chosen per animal. Image J (v1.48, http://imagej.nih.gov/ij/, National Institute of Health, Bethesda, MD, USA) was applied to measure the length of placenta using a straight line tool. The area measurements were performed using a threshold-based method. Placental data were obtained at the middle level. Fetal brain data were collected from the cortical area, including the cortex plate, subplate, intermediate zone, and ventricular zone.

### Fibrinogen and fibrin measurement

Fibrinogen levels can be used as an indirect measure for the consumption of clotting factors following thrombus formation. Fibrinogen and fibrin in placenta were measured using an enzyme-linked immunosorbent assay (ELISA) kit purchased commercially (Abcam, Cambridge, MA, USA). Following the manufacturer’s protocol, the plates were read using the CLARIOstar system (BMG Labtech, Cary, NC, USA), and final data were normalized by protein concentration. Histochemical staining (rapid phosphotungstic acid hematoxylin, PTAH, Polyscience Inc, Warrington, PA, US) was applied to the placenta for fibrin staining, according to the manufacturer's protocol.

### Statistical analyses

The US-based indices, MRI-based placental volumetric measurements, H&E-based placental thickness measurements, and fibrinogen/fibrin levels in the LPS and PBS groups were compared using two-tailed Student’s t-tests with unequal variance. Vimentin and Iba1 positive expression were determined through quantitative analyses; specifically, the vascular density was obtained using positive area divided by total area, and means of the measures were compared using the Student’s t-test. Placental thickness was correlated with microglial activation (Iba1 expression) using the Pearson correlation analysis (Graphpad Prism). The statistically significant differences were recognized as p<0.05.

## Results

### Uterine and umbilical artery insufficiency in placentas and the left ventricle Tei index increased in fetuses exposed to IUI

**Fig 1:**
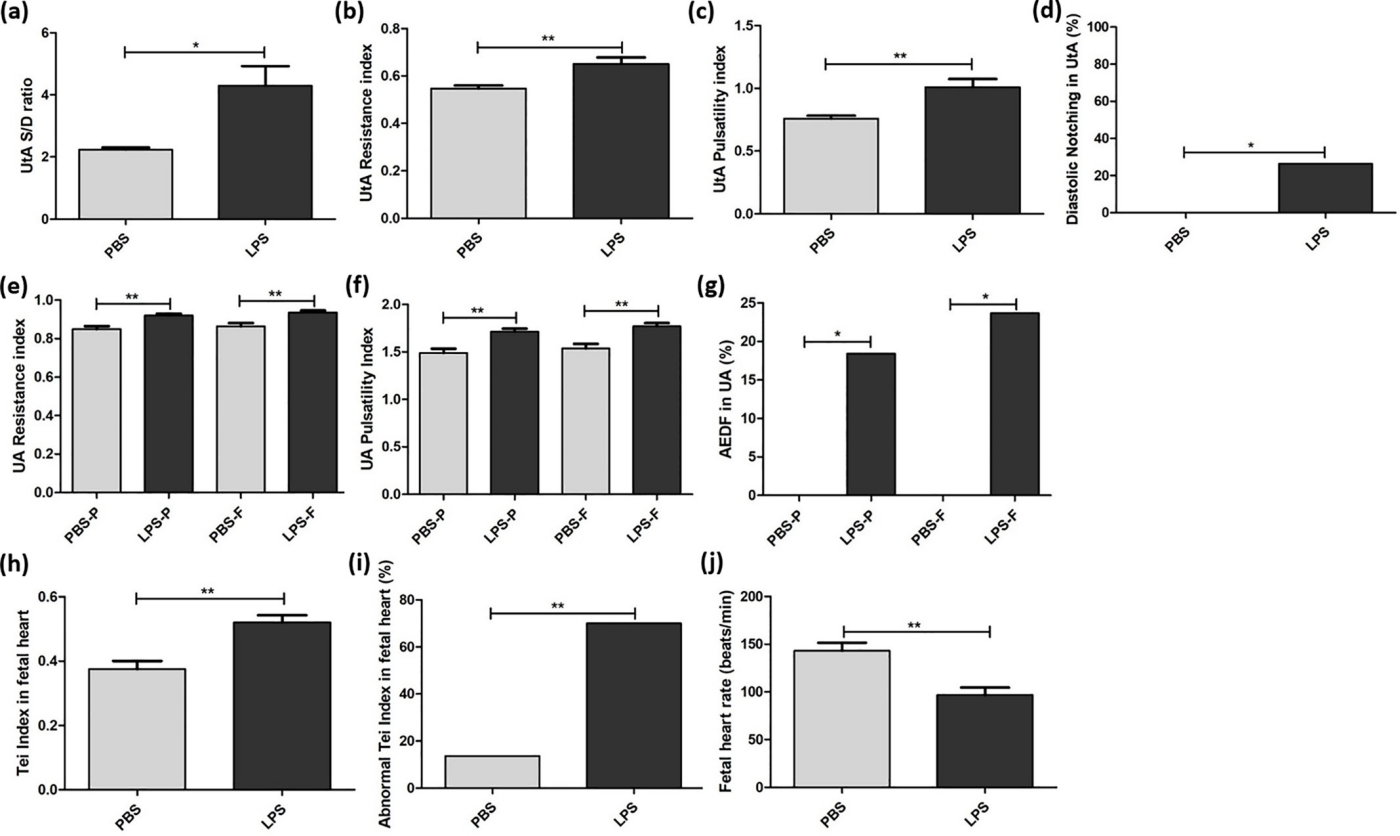
Uterine and umbilical artery insufficiency and abnormal Tei index of the fetal heart exposed to intrauterine inflammation. Doppler waveforms were interrogated six hours after phosphate-buffered saline (PBS) or lipopolysaccharide (LPS) exposure. Doppler was used to assess the uterine artery (UtA), placental side of the umbilical artery (UA) and fetal side of the UA in the PBS group, as well as the UtA, placental side of the UA and fetal side of the UA in the LPS group. (a) The systolic/diastolic (S/D) ratio, (b) resistance indices, (c) pulsatility indices and (d) rate of early diastolic notch in UtA were determined for the LPS group (n = 17 dams, 34 arteries) and the PBS group (n = 8 dams, 16 arteries). (e) Resistance indices, (f) pulsatility indices and (g) the rate of absent end-diastolic flow (AEDF) were found for both the placental (-P) and fetal (-F) sides of the UA in the LPS group (n = 17 dams, 38 pups) and the PBS group (n = 8 dams, 26 pups). Time intervals of Tissue Doppler for evaluating Tei indices are shown for the PBS group and the LPS group. (h) Tei indices, (i) the rate of abnormal Tei index (>0.44) and (j) fetal heart rates were compared for the LPS group (n = 17 dams, 40 pups) and the PBS group (n = 8 dams, 22 pups). *p<0.05, **p<0.01.

PBS- and LPS-exposed groups exhibited significant differences in Doppler waveforms at the UtA and both the placental and fetal sides of the UA. Specifically in the UtA, the S/D ratio (2.24 ± 0.28 vs. 4.30 ± 3.68, p<0.05), RI (0.55 ± 0.05 vs. 0.65 ± 0.16, p<0.01), PI (0.76 ± 0.10 vs. 1.01 ± 0.38, p<0.01) and rate of early diastolic notch (0.0% vs. 26.5%, p<0.05) were significantly higher in the LPS group compared to the PBS group. The RI (0.85 ± 0.08 vs. 0.92 ± 0.06, p<0.01), PI (1.49 ± 0.23 vs. 1.71 ± 0.19, p<0.01) and rate of AEDF (0.0% vs. 18.4%, p<0.05) in the placental side of the UA, as well as the RI (0.86 ± 0.08 vs. 0.94 ± 0.06, p<0.01), PI (1.54 ± 0.23 vs. 1.77 ± 0.21, p<0.01) and rate of AEDF (0.0% vs. 23.7%, p<0.05) in the fetal side of the UA, were significantly higher in the LPS group compared to the PBS group (**Fig 1A–1G**).

Tei indices were significantly increased in the LPS group compared to the PBS group (0.37 ± 0.12 vs. 0.52 ± 0.14, p<0.01). The rate of abnormal Tei indices (>0.44) was higher in the LPS group compared to the PBS group (13.6% vs. 70.0%, p<0.01) (**Fig 1H–1J**). Considering the significant difference in fetal heart rates between the two groups (143 ± 40 vs. 97 ± 50, p<0.01), a multivariate analysis was performed. The rate of abnormal Tei indices was still higher in the LPS group than the PBS group (adjusted odds ratio 18.24, 95% CI 3.62–91.84).

### Decreased volume and thickness of placentas exposed to IUI

**Fig 2:**
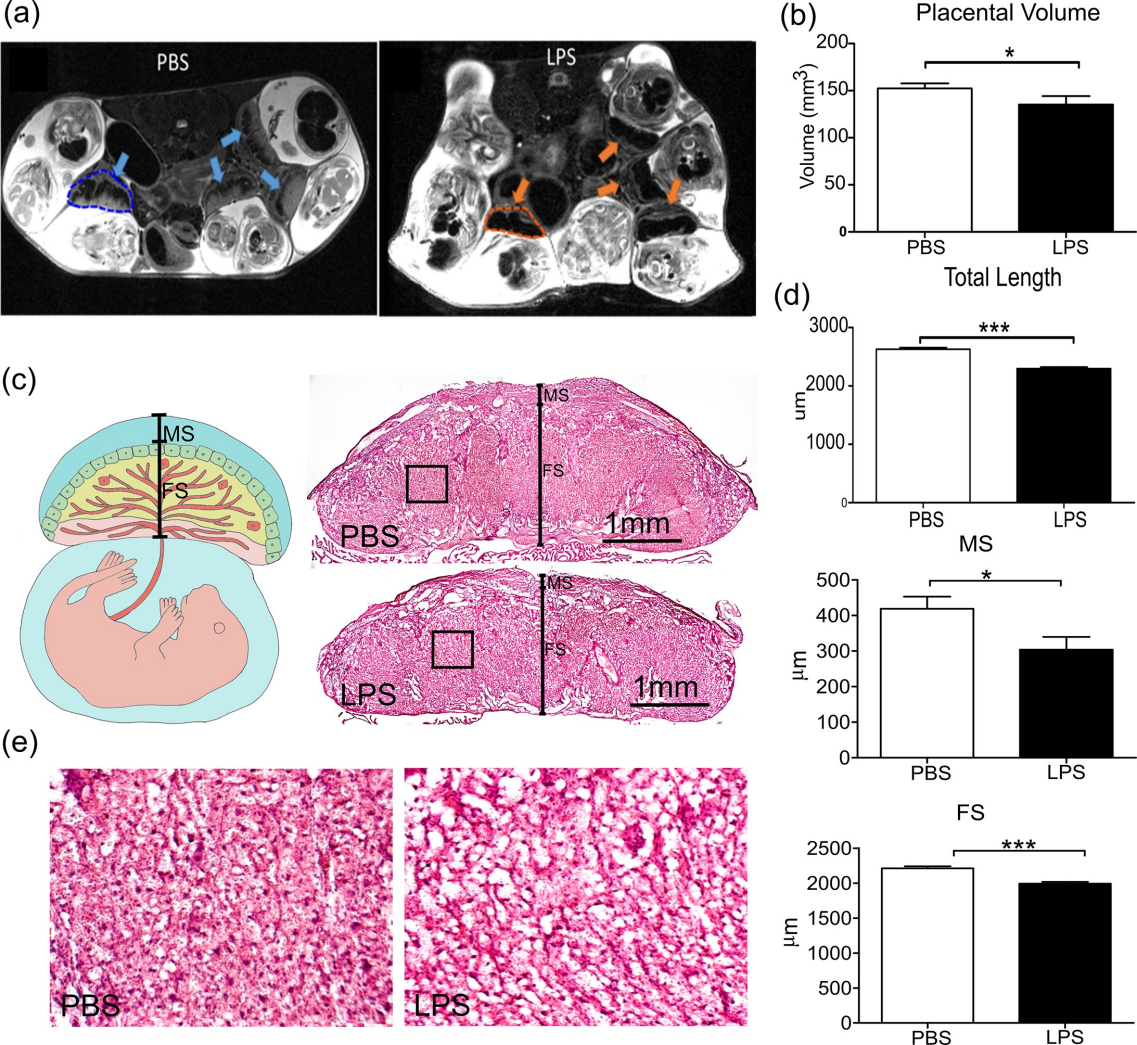
MRI imaging and analysis, revealing decreased volume and thickness of placentas exposed to intrauterine inflammation. *(a)* T2-weighted images of placentas in mice treated with phosphate-buffered saline (PBS) or lipopolysaccharide (LPS) were obtained. The blue and orange arrows note placental locations, while the dashed contours indicate examples of the manual delineation of the placentas (performed in 3D) for volumetric analysis. *(b)* 3D measurements shows the individual placenta volumes from the PBS (n = 15) and LPS (n = 14) groups. *(c)* Hematoxylin and eosin (H&E) stains were performed to compare LPS (n = 25) and PBS (n = 20) placental thicknesses at 5X magnification. The solid black line crossing the central cut surface of the placentas indicated the location of the measurements. The maternal segments (MS)–decidual zone–and fetal segments (FS) were identified as a compilation of junctional zone and labyrinth. *(d)* Statistical analysis shows the results of the H&E stain measuring maternal, fetal and total placental length at their thickest levels. *(e)* The images corresponded to the black boxes in *(c)* with high magnification. *p<0.05; ***p<0.001.

The mean volume of LPS-exposed placentas was significantly smaller than that of controls with a 13% decrease (**Fig 2A and 2B**, 155.3 ± 4.808 vs. 134.4 ± 8.746, p<0.05, two-tailed Student’s t-test with unequal variances). Additionally, H&E staining showed significantly reduced thickness in the LPS-exposed placentas compared to the PBS-exposed ones (**Fig 2C and 2D**, 2630 ± 26.47 vs. 2296 ± 29.08, p<0.0001, two-tailed Student’s t-test with unequal variances), with placental thinning found in both maternal and fetal components of LPS-exposed placentas.

### Decreased CD31 and vimentin in placentas exposed to IUI

**Fig 3:**
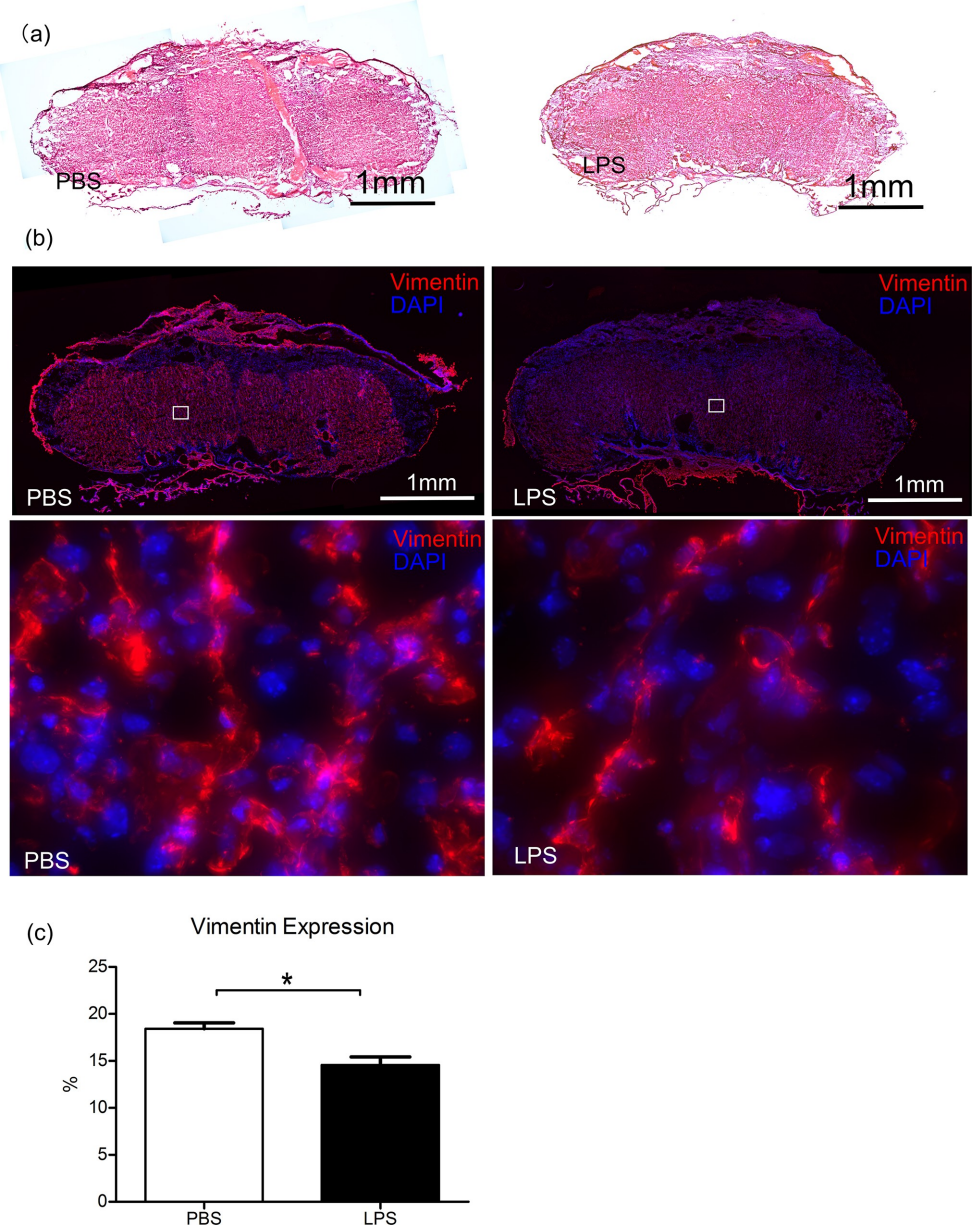
Vimentin staining of placentas, indicating the endothelial cells. *(a)* Hematoxylin and eosin (H&E) stains the neighbor sections indicates the central cut surface of the placentas. *(b)* Immunohistochemistry staining of vimentin (red) was shown for placentas exposed to phosphate-buffered saline (PBS, n = 5) or lipopolysaccharide (LPS, n = 5). DAPI (blue) stands for counter-staining of nuclei. The bottom panel is the magnification of the inserts in top panel. *(c)* Quantitative measurements show the percentage of vimentin expression in PBS and LPS placentas. *p<0.05.

IHC staining for CD31[17] and vimentin [18], endothelial cell markers, demonstrated less CD31 and vimentin expression in both the maternal and fetal segments of LPS-exposed placentas (**Fig 3B & S1 Fig)**. Quantitative measurements indicated that the positive CD31 (20.74 ± 0.64 vs. 16.38 ± 0.97) and vimentin (18.43 ± 0.62 vs. 14.58 ± 0.84) expression areas in the LPS group were significantly lower than that in the PBS group (**Fig 3C,** p<0.01, two-tailed Student’s t-test).

### Decreased fibrinogen in placentas exposed to IUI

**Fig 4:**
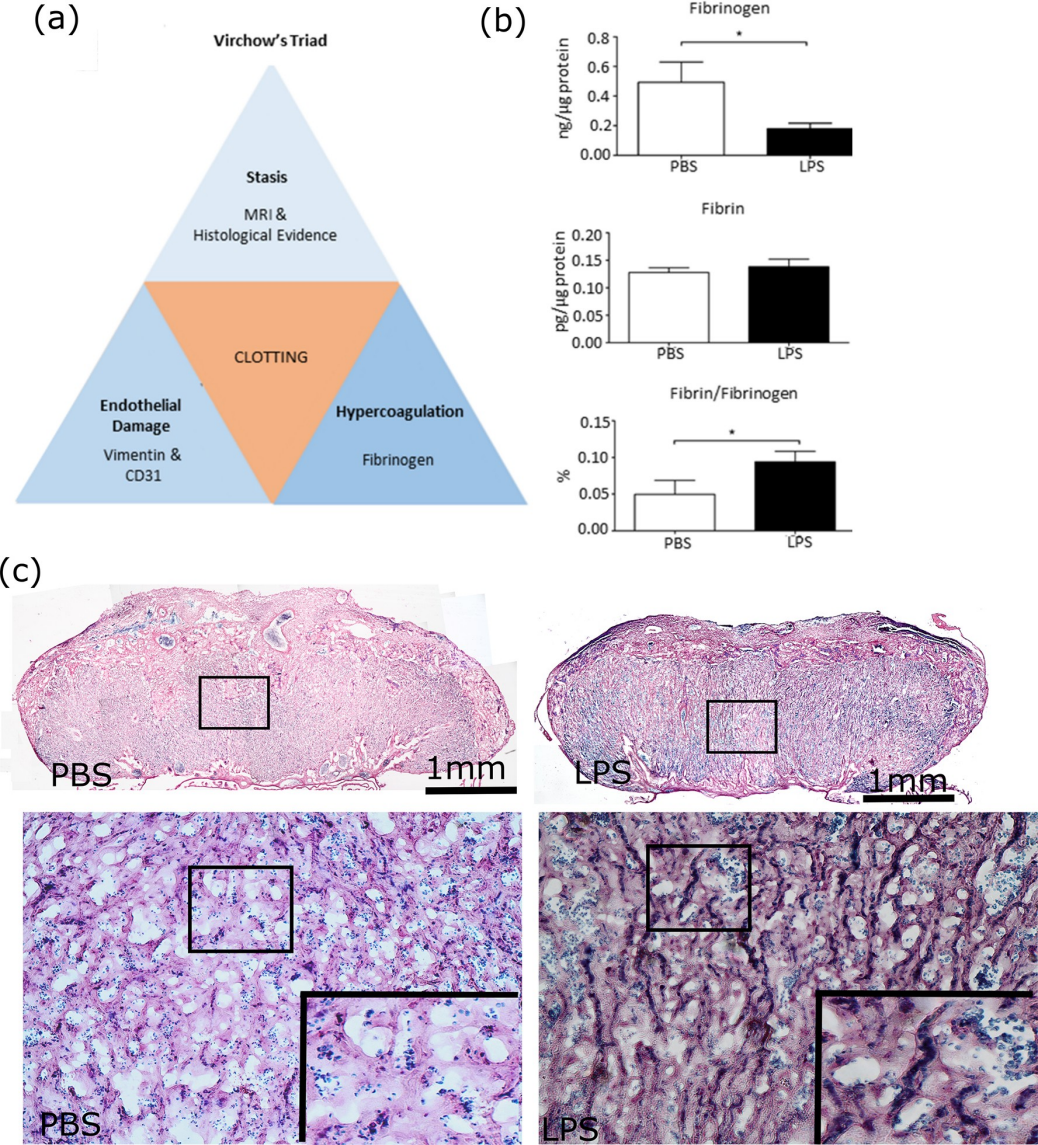
Virchow’s Triad, including hypercoagulability suggested by decreased fibrinogen in placentas exposed to intrauterine inflammation. *(a)* This schematic illustrates the three primary factors that contribute to clot formation, according to Virchow’s triad, as well as the lab techniques used to verify their presence in our study mice. *(b)* The graph shows the fibrinogen levels detected by enzyme-linked immunosorbent assay (ELISA) in placentas exposed to phosphate-buffered saline (PBS, n = 7) or lipopolysaccharide (LPS, n = 8). *(c)* Histochemical staining of placentas for fibrin show abundant fibrin-rich thrombi (dark blue) in the villi of placenta exposed to LPS. The inserts were the high magnification of the black box in the same panel. *p<0.05.

ELISA with quantitative analysis revealed significantly less fibrinogen (0.1798 ± 0.03762 vs. 0.4931 ± 0.1370), comparable amounts of fibrin (0.1383 ± 0.0138 vs. 0.1278 ± 0.0087) and a significantly smaller percentage of fibrin to fibrinogen (0.0940 ± 0.0143 vs. 0.04980 ± 0.0189) in placentas exposed to IU LPS versus PBS (**Fig 4B**, p<0.05, two-tailed Student’s t-test). These results indicate a consumption of clotting factor with the formation of thrombi. Histochemical staining revealed fibrin-rich thrombi (dark blue) in some villi of placenta exposed to maternal LPS (**Fig 4C**).

### Iba1 expression increased in brains of fetuses exposed to IUI

**Fig 5:**
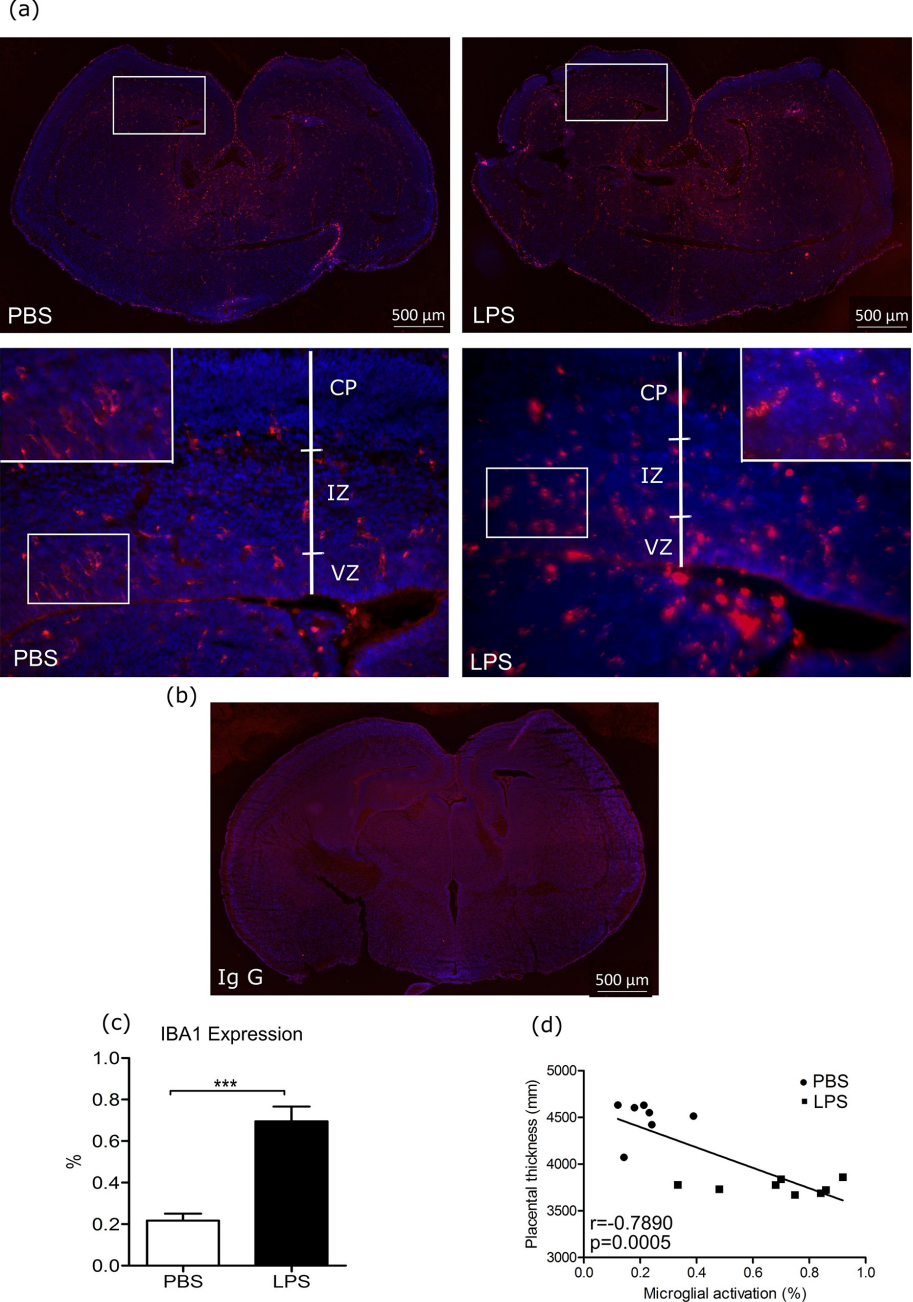
Iba 1 expression in fetal brain and correlation to placental thickness. *(a)* Representative images of ionized calcium-binding adaptor molecule 1 (Iba1) expression in fetal brain of E17 mice six hours after lipopolysaccharide (LPS) intra-uterine exposure are presented, the studied areas were the cortical area, including the cortex plate, intermediate zone, and ventricular zone. *(b)* Negative control image using rabbit Ig G in place of primary antibody. *(c)* Iba1 positive expression in fetal brains was determined through quantitative analysis as the percentage of Iba1 expression area within the image using Image J (PBS: n = 7, LPS: n = 8). *(d)* Placental thickness was correlated with microglial activation (Pearson correlation analysis). A Panel: blue represents DAPI stain for nuclei, and red represents Iba1 stain. *** p<0.001; scale bar: 50μm. CP: cortical plate, IZ: intermediate zone, VZ: ventricular zone.

Images representative of Iba1 expression in the fetal brain following intrauterine exposure to LPS and PBS were selected (**Fig 5A**). Iba1 expression was significantly increased in fetal brains exposed to LPS as compared to PBS (**Fig 5C,** 0.6955 ± 0.0704 vs. 0.2171 ± 0.0333, p<0.001, Student’s t-test). Additionally, placental thickness was negatively correlated to the percentage of microglial activation (**Fig 5D,** p<0.001, r = -0.7890, Pearson correlation analysis).

## Discussion

Utilizing a mouse model of IUI, for the first time, we evaluated if maternal and fetal malperfusion following exposure to IUI were associated with fetal sequelae (changes in cardiac Tei indices and fetal neuroinflammation). Consistent with other MRI studies[19,20], showing the association between the placental insufficiency and inflammatory pathway, our data demonstrate US-detectable hemodynamic changes in uterine and umbilical blood flow, which are confirmed by changes in placental volume and associated with endothelial damage and thrombi formation (decrease in fibrinogen). Most importantly, we demonstrate that placental thinning following IUI is associated with fetal microglial activation (r = -0.7890, Pearson correlation coefficient).

Virchow’s triad asserts that hypercoagulability (as in pregnancy), injury to vasculature, and a disruption or interruption in blood flow collectively contribute to the development of thrombosis [21]. In our study, we demonstrate placental endothelial damage through vimentin and CD31 staining, as well as thrombosis through fibrinogen analysis. We speculate that the placental thinning, with concomitant damaged vasculature following IUI exposure may result in stagnant blood flow–the third component of Virchow’s triad. Consequently, we propose that fetal microglial activation (a sign of fetal brain inflammation) may be associated with a placental thrombotic disruption in the passage of nutrients, gases, and toxins across the placenta and, hence, be a result of hypoxic-ischemic injury.

Studies have previously demonstrated that thrombi in the vasculature of the fetal side of the placenta were associated with severe injury to neonates, and the fetal brain seems particularly vulnerable to thrombotic disease based on its significant and constant receipt of circulating blood [22,23]. Specifically, Kraus and Acheen revealed that approximately one third of stillborns with extensive fetal thrombotic vasculopathy (FTV) in the placenta were also shown to suffer from thrombi in fetal organs, including the brain [23]. Moreover, the detection of thrombi in the fetal vasculature of the placenta has been linked to cerebral palsy in infants (18), whereas Lepais, Gaillot-Durand, Boutitie et al. observed a greater–though not significant–incidence of neurodevelopmental and language delays, as well as behavioral and motor disorders, in children whose placentas exhibited FTV lesions[24].

Maternal placental malperfusion is known to lead to abnormal uterine blood flow [25,26,27,28,29]. While malperfusion in the fetal compartment has the same effect on the umbilical waveform, fetuses exposed to such abnormalities are at risk for more widespread metabolic and cardiovascular sequelae, which have been linked to brain injury [30]. Furthermore, increased velocimetry indices are known to be concerning for fetal hypoxia and are associated with adverse perinatal outcomes, such as preeclampsia, preterm birth, fetal growth restriction (FGR), and increased cardiovascular and central nervous system problems in newborns[31,32,33,34,35]. Corollary to that, in our study, we demonstrate changes in Tei indices and fetal neuroinflammation following the observed changes in the fetoplacental hemodynamics.

The Tei index has mainly been used in amyloidosis, dilated cardiomyopathy, ischemic heart disease, and congestive heart failure; it is considered a useful predictor of global cardiac function, independent of both heart rate and blood pressure[36,37]. Some studies suggest that an increase in the Tei index may indicate ventricular dysfunction in fetuses with FGR, preeclampsia, and diabetes [38,39,40,41]. In clinical studies, the index has been used as an indicator of ventricular dysfunction in fetuses exposed to IUI [42]. Furthermore, fetuses suffering from FIRS who are unable to change cardiac compliance and exhibit worsening brain perfusion could develop fetal periventricular leukomalacia[43]. Taken together with our animal studies, the Tei index may be a useful predictor of fetal sequelae in cases suspicious for IUI exposure, such as preterm premature rupture of membranes (pPROM). Further clinical studies are needed to validate this tool. Furthermore, future research is needed to demonstrate whether heart dysfunction as a result of placental malperfusion may play a key role in the fetal neuroinflammation.

Although most prior studies examining UA Doppler indices have focused on placenta-based FGR, we speculate that this knowledge can be applied to identify patients at risk for developing sequelae of IUI. Our study is the first to document that IUI following intrauterine administration of LPS (mimicking the most common clinical scenario associated with preterm birth) leads to fetoplacental hemodynamic changes. Our study also highlights the potential importance of the level of interrogation of the UA along the umbilical cord where the sampling occurs.

Our study is supported by our prior work investigating MRI-based placental perfusion deficits following the exposure to IUI [44]. Specifically, we used MRI to examine placental damage following IUI and found a significant decrease in capillary blood volume and velocity, suggestive of vascular damage and blood flow disruption [44,45,46]. We speculate that these effects may contribute to the formation of a clot, subsequently decreasing placental volume. The results of our current study add further support to this speculation and correlate the acute placental injury following IUI to fetal sequelae [45].

Furthermore, our fetal MRI work previously demonstrated that fetal cortical injury could be detected from apparent diffusivity coefficient maps as early as six hours after exposure to inflammation and, in clinical studies, placental volumes are correlated with fetal brain volumes [44,46]. Taken together, US with or without concomitant MRI may serve as a useful clinical tool for delineating normal and pathological placentas, as well as predicting fetal sequelae in such cases of IUI exposure as preterm labor, pPROM, chorioamnionitis, FIRS, and potentially even thrombotic injuries.

In conclusion, our data provide evidence that IUI can trigger a sequence of events leading to acute maternal placental malperfusion and predispose the affected fetus to cardiac dysfunction and brain inflammation. The combination of malperfusion and potential effects of placental thickness appear to be associated with fetal microglial activation. In this setting, further clinical investigations are necessary to establish the role of Doppler US as a clinical tool used to indirectly assess fetuses at risk for adverse neurologic outcomes following an exposure to IUI (e.g. preterm labor and pPROM).

## Supporting information

S1 Fig CD31 staining of placentas, indicating the endothelial cells. *(a)* Immunohistochemistry staining of CD31 (green) was shown for placentas exposed to phosphate-buffered saline (PBS, n = 5) or lipopolysaccharide (LPS, n = 5). DAPI (blue) stands for counter-staining of nuclei. *(b)* Quantitative measurements show the percentage of CD31 expression in PBS and LPS placentas. *p<0.05.
(DOCX)
