# Supplementary material for: Placental malperfusion in response to intrauterine inflammation and its connection to fetal sequelae
Source: PLoS One. 2019 Apr 3;14(4):e0214951. doi: 10.1371/journal.pone.0214951 (PMC6447225; doi:10.1371/journal.pone.0214951)
Supplement: S1 Fig — (a) Immunohistochemistry staining of CD31 (green) was shown for placentas exposed to phosphate-buffered saline (PBS, n = 5) or lipopolysaccharide (LPS, n = 5). DAPI (blue) stands for counter-staining of nuclei. (b) Quantitative measurements show the percentage of CD31 expression in PBS and LPS placentas. *p<0.05. (DOCX) [file pone.0214951.s001.docx]

**Placental malperfusion in response to intrauterine inflammation and its connection to fetal sequelae**

Solange N. Eloundou^1,^, JiYeon Lee^1,^, Dan Wu^2^, Jun Lei^1^, Mia C. Feller^1^, Maide Ozen^1,3^, Yan Zhu^1^, Misun Hwang^2^, Bei Jia^1^, Han Xie^1^, Julia L. Clemens^1^, Michael W. McLane^1^, Samar AlSaggaf^4^, Nita Nair^1^, Marsha Wills-Karp^5^, Xiaobin Wang^6^, Ernest M. Graham^1^, Ahmet Baschat^7^, Irina Burd^1*^

^1^ Integrated Research Center for Fetal Medicine, Division of Maternal Fetal Medicine, Department of Gynecology and Obstetrics, Johns Hopkins University, School of Medicine, Baltimore, MD, 21287, USA

^2^ The Russell H. Morgan Department of Radiology and Radiological Science, Johns Hopkins University, School of Medicine, Baltimore, MD, 21287, USA

^3^ Division of Neonatology, Department of Pediatrics, Johns Hopkins University, School of Medicine, Baltimore, MD, 21287, USA

^4^ Department of Pathology, King Abdulaziz University, Jeddah, 21589, Kingdom of Saudi Arabia

^5^ Department of Environmental Health and Engineering, Johns Hopkins University, School of Public Health, Baltimore, MD, 21205, USA

^6^ Department of Population, Family and Reproductive Health, Center on Early Life Origins of Disease, Johns Hopkins University, School of Public Health, Baltimore, MD, 21205, USA

^7^ Fetal Therapy, Department of Gynecology and Obstetrics, Johns Hopkins University, School of Medicine, Baltimore, MD, 21287, USA

*Corresponding author

E-mail: iburd@jhmi.edu


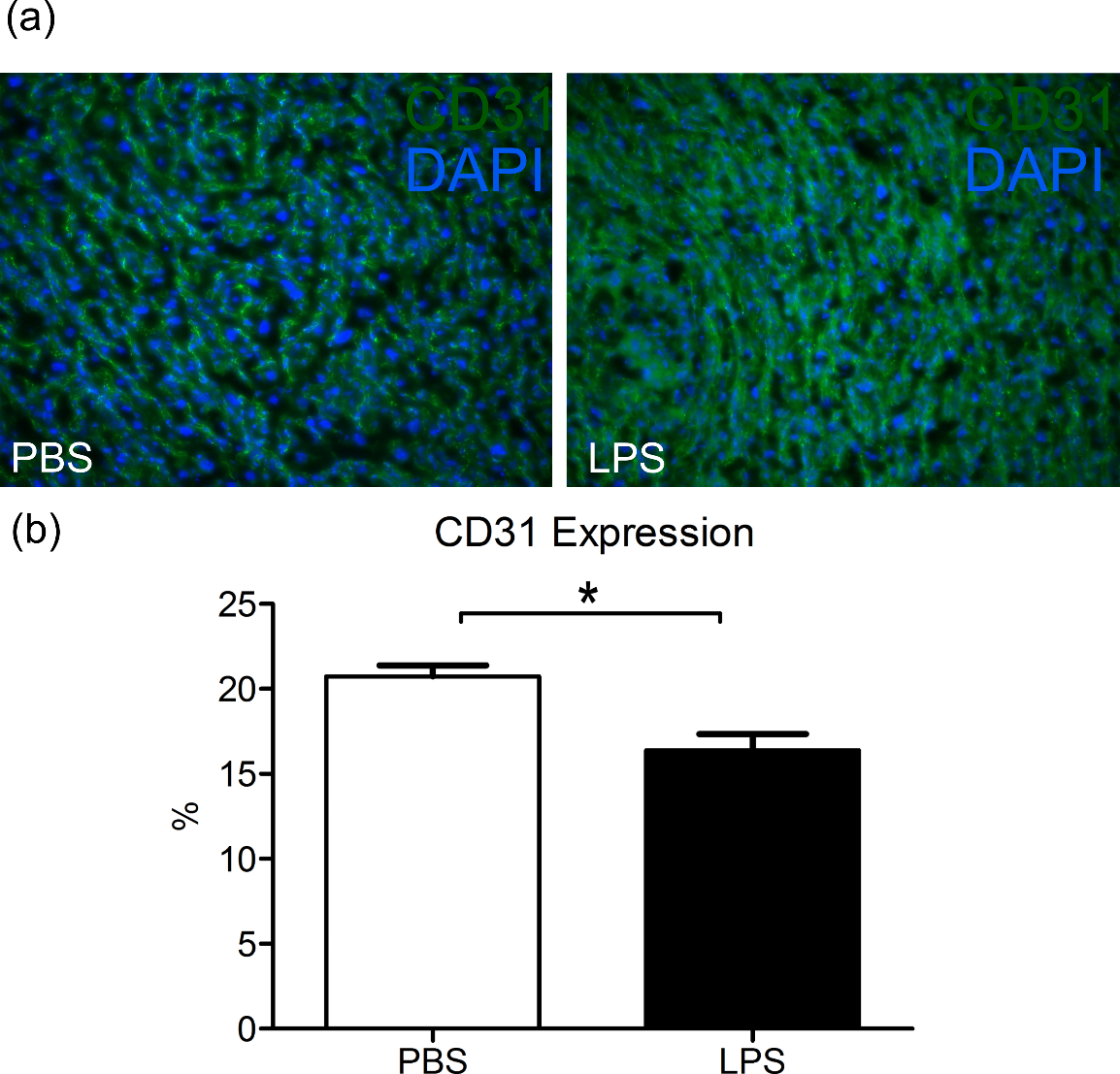


**S1 Fig. CD31 staining** **of placentas, indicating the endothelial cells.**

*(a)* Immunohistochemistry staining of CD31 (green) was shown for placentas exposed to phosphate-buffered saline (PBS, n=5) or lipopolysaccharide (LPS, n=5). DAPI (blue) stands for counter-staining of nuclei. *(b)* Quantitative measurements show the percentage of CD31 expression in PBS and LPS placentas. *p<0.05.
